# Retrospective analysis of risk indicators for erosive tooth wear in patients from Midwest US

**DOI:** 10.1007/s00784-026-06981-x

**Published:** 2026-06-13

**Authors:** Danielle C Messias, Ariene A Leme-Kraus, Stephanie E Lewis, Erica C Teixeira

**Affiliations:** 1 Indiana University School of Dentistry, 1121 W Michigan St, Indianapolis, IN 46202 USA; 2 The University of Iowa College of Dentistry and Dental Clinics, 801 Newton Rd, Iowa City, IA 52242 USA; 3 Texas A&M University College of Dentistry, 3302 Gaston Ave., Dallas, TX 75246 USA

**Keywords:** Erosive tooth wear, Risk indicators, Age, Sex, Parafunctional habits, Eating disorder

## Abstract

**Objectives:**

This retrospective study aimed to identify risk indicators for erosive tooth wear (ETW) in patients from the Midwest US.

**Materials and methods:**

Clinical records from 2019 to 2023 were reviewed from 2,482 adult patients diagnosed with ETW at the University of Iowa College of Dentistry. A control group of 2,967 patients without ETW was randomly selected from the same timeframe. Data on demographic information, self-reported medical history, and oral health condition/habits were extracted. Logistic regression analysis (α = 0.05) was used to evaluate associations between ETW and potential risk indicators.

**Results:**

ETW was significantly associated with age (35–54 years: OR = 1.24; *p* = 0.03), male sex (OR = 1.43; *p* < 0.001), parafunctional habits (OR = 1.44; *p* < 0.001), and eating disorders (OR = 1.90; *p* = 0.014). No significant associations were found for Social Deprivation Index (SDI), sleep apnea, dry mouth, toothbrushing frequency, alcohol use, or GERD.

**Conclusions:**

Within the limitations of this retrospective study, age (35–54 years), male sex, parafunctional habits (clenching, grinding or bruxism), and eating disorders (bulimia and/or anorexia nervosa) were identified as significant risk indicators for erosive tooth wear. Recognition of these factors may support earlier diagnosis and tailored preventive strategies.

**Clinical relevance:**

Dental professionals should be particularly attentive to patients aged 35–54 years, males, and those with parafunctional habits or eating disorders, as they are more likely to present with erosive tooth wear. Early identification of these risk indicators can guide preventive counseling and individualized management to reduce the progression and long-term impact of the condition.

## Introduction

Erosive tooth wear (ETW) is a multifactorial condition characterized by the progressive loss of dental hard tissue due to chemical dissolution by non-bacterial acids. The loss of dental structure is believed to be exacerbated by mechanical forces such as abrasion and attrition, and the interaction between erosive and abrasive processes is recognized as a key driver of accelerated tissue loss [1]. Unlike caries, the acids responsible for ETW originate from intrinsic sources, such as gastric juice reaching the mouth during reflux or vomiting [2], and extrinsic sources, including acidic foods and beverages, and environmental exposure [3]. Dietary factors, particularly the frequent consumption of acidic drinks and foods, have been consistently identified as major contributors to ETW progression [3]. If left untreated, ETW can lead to extensive loss of dental structure causing hypersensitivity, compromised aesthetics, and impaired oral function. These can significantly affect patients’ quality of life [4] and increase long-term treatment costs for both patients and the healthcare system [5].

Globally, ETW has become increasingly prevalent, with studies reporting rates ranging from 20% to 45% in permanent dentition among adults [6]. Risk groups, including individuals with eating disorders, gastroesophageal reflux disease (GERD), high consumption of acidic beverages, and certain occupational exposures, show markedly elevated prevalence rates, often exceeding 50% [7]. Despite this growing concern, epidemiological data from the United States remain limited [6], fragmented, and regionally underrepresented, making it difficult to assess the true burden of ETW across diverse American populations. The absence of large-scale, population-based U.S. studies represents a critical gap in the literature, as findings from other countries may not be directly applicable. The clinical assessment of ETW has been standardized through validated indices such as the Basic Erosive Wear Examination (BEWE), which provides a reproducible and clinically applicable scoring system for the graded assessment of erosive wear [8]. However, the variability in diagnostic criteria adopted across epidemiological studies contributes to the wide range of prevalence estimates reported in the literature and complicates direct comparisons across populations.

The prevalence and contributing factors of ETW can vary significantly across countries and regions due to differences in diet, cultural habits, socioeconomic conditions, and healthcare access [9]. Recognizing these local realities is essential for clinicians and public health professionals to effectively identify at-risk individuals and tailor preventive strategies. In particular, understanding the sociodemographic and behavioral context of a given population allows for more targeted oral health promotion, early diagnosis, and timely intervention.

The aim of this retrospective study was to explore the association between the occurrence of ETW and potential risk indicators, including sociodemographic factors, health and oral conditions, oral hygiene practices, and lifestyle habits, among patients from the Midwest U.S. treated at the dental clinic of the University of Iowa College of Dentistry. Given the multifactorial aetiology of ETW and the limited epidemiological evidence available in this clinical population, this study was designed as an exploratory investigation intended to identify factors potentially associated with ETW occurrence and to generate hypotheses for future confirmatory research.

## Materials and methods

This retrospective case-control study included patients seen at the dental clinics of the University of Iowa College of Dentistry, Iowa City, USA, from 2019 to 2023. Ethical approval was obtained from the Institutional Review Board of the University of Iowa.

Eligible participants were dentate patients aged ≥ 18 years who had undergone a comprehensive examination. Cases were defined as all patients diagnosed with ETW, presenting at least one erosive tooth lesion during the study period. Controls were selected as a sample of patients without ETW from the same source population, defined as having no teeth with erosive lesions.

### Data collection

For data collection, electronic dental records (axiUm) from the University of Iowa College of Dentistry and Dental Clinics were accessed. All patients diagnosed with at least one tooth with ETW during the period between 2019 and 2023 were selected. ETW was identified in patients’ records by the diagnostic code for dental erosion and/or through records notes, using the keywords “erosion”, “dental erosion”, “erosive tooth wear”, and respective misspellings. These records were based on clinical findings gathered during the patient’s comprehensive examination and recorded in the tooth chart during clinical appointments by dental students (pre-doctoral and graduate) and faculty providers. In routine clinical practice, diagnoses of ETW lesions were based on clinical judgment, informed by the presence of clinical signs consistent with erosive tooth wear — including enamel surface loss, cupping, dentin exposure, and surface flattening — as well as differential diagnosis and relevant information obtained from the patient’s medical history and anamnesis. All clinical assessments were conducted under supervising faculty oversight. For the sample without ETW, 2,967 subjects were randomly selected as controls from a larger eligible pool presenting during the same timeframe (2019–2023). No matching procedure was performed between cases and controls. The index date was defined as the date of the first ETW registration for cases and the date of the last clinical visit for controls.

All recognized variables were extracted from patients’ records and health history forms (Table 1). Except for age and Social Deprivation Index (SDI), all health and behavioral variables were self-reported by the patients.

**Table 1:** Variables considered in the study

Category	Variable	Description / Classification
**Sociodemographic**	Age	Age at diagnosis (ETW) or last visit (non-ETW); grouped as: 18–34, 35–54, ≥ 55 years
	Sex	Male or Female
**Socioeconomic**	SDI*	Grouped into percentiles: ≤25th, 26th–50th, 51st–75th, > 75th
General Health	Gastroesophageal reflux (GERD)	Yes or No
	Eating disorder	Bulimia and/or anorexia nervosa; Yes or No
	Sleep apnea	Yes or No
Oral Health	Dry mouth	Yes or No
	Parafunctional habits	Clenching, grinding or bruxism; Yes or No
Oral Hygiene	Toothbrushing frequency	At least once daily or twice or more daily
Medication	Saliva-reducing medication†	Yes or No
Lifestyle Behavior	Alcohol consumption	Yes or No

* SDI values were assigned based on ZIP Code using the 2019 Social Deprivation Index dataset

† Saliva-reducing medications include tranquilizers, antipsychotics, antihistamines, cortisol inhalers, and diuretics

‡ All health and behavioral variables were self-reported by patients at intake or during follow-up visits

All self-reported data, containing “don’t know” as a response, were relabeled as missing data prior to the statistical analysis.

### Statistical analysis

For data analysis, each patient was accounted for once. Given the possibility that a single patient may have undergone multiple comprehensive exams, individuals considered not diagnosed with ETW never had the diagnostic code or the specific terms mentioned in any exam note. For patients diagnosed with ETW who had multiple diagnostic code registrations, the first registration of the ETW was considered to be the time of diagnosis.

To assess the effect of the risk indicators on ETW, a multivariable logistic regression model was used with ETW as the outcome. The multivariable logistic regression model was fitted using complete cases only; patients with missing values in any covariate included in the model were excluded from the regression analysis. Table 2 displays the summary statistics predictor variables in the model. Age and SDI were categorized according to clinically relevant cut points, and brushing frequency was dichotomized according to the distribution of the data and clinical interest. Due to the large sample size and study goals of interpretation rather than prediction, no model/variable selection methods were used. Likewise, there were no interaction effects of clinical interest, so interactions were not considered in the model. Standard logistic regression diagnostics of multicollinearity checking and influential values were employed to ensure model validity. All analyses were done using R version 4.3.1 (2023-06-16 ucrt), at the significance level of 0.05.

**Table 2:** Descriptive summary of the sample

Risk indicators		No ETW = 2967	ETW = 2482
Age	18–34 years	960 (32%)	684 (28%)
	35–54 years	812 (27%)	795 (32%)
	≥ 55 years	1195 (40%)	1003 (40%)
Sex (missing: C = 14, ETW = 19)	female	1657 (56%)	1271 (52%)
	male	1296 (44%)	1192 (48%)
SDI (missing: C = 7, ETW = 9)	≤ 25th	789 (27%)	613 (25%)
	26th – 50th	788 (27%)	709 (29%)
	51st – 75th	1069 (36%)	860 (35%)
	> 75th	314 (11%)	291 (12%)
GERD (missing: C = 224, ETW = 121)	Yes	545 (20%)	575 (24%)
	No	2198 (80%)	1786 (76%)
Eating Disorder (missing: C = 225, ETW = 116)	Yes	34 (1.2%)	66 (2.8%)
	No	2708 (99%)	2300 (97%)
Sleep apnea (missing: C = 235, ETW = 135)	Yes	212 (7.8%)	207 (8.8%)
	No	2520 (92%)	2140 (91%)
Dry mouth (missing: C = 540, ETW = 348)	Yes	734 (30%)	691 (32%)
	No	1693 (70%)	1443 (68%)
Parafunctional habits (missing: C = 688, ETW = 456)	Yes	859 (38%)	978 (48%)
	No	1420 (62%)	1048 (52%)
Toothbrushing frequency (missing: C = 562, ETW = 364)	at least once daily	1066 (44%)	867 (41%)
	twice or more daily	1339 (56%)	1251 (59%)
Medication (missing: C = 752, ETW = 527)	Yes	1181 (53%)	1056 (54%)
	No	1034 (47%)	899 (46%)
**Dallas** (missing: C = 228, ETW = 115)	Yes	1095 (40%)	1060 (45%)
	No	1644 (60%)	1307 (55%)

*C* control group, *ETW* erosive tooth wear group

## Results

A total of 2482 dental records with ETW and 2967 without ETW registration were included in the study. Table 2 presents the characteristics of the full sample.

Of the 5449 subjects described in Tables 2, 3089 had complete data (e.g., no missing information in any variable). Of these, 1456 had ETW and 1633 did not. These complete cases were used to construct the multivariable logistic regression model. The model (Table 3) showed that age, sex, self-reported eating disorder, and parafunctional habits were significantly associated with ETW. Patients in the age group between 35 and 54 years had 24.2% increased odds of presenting ETW compared to those aged 18–34. Males presented 43.5% higher odds of ETW than females. Those reporting an eating disorder (bulimia and/or anorexia) had 90.1% increased odds of presenting ETW compared to those that did not report the same condition. Patients who reported parafunctional habits (clenching, grinding or bruxism) had 43.6% increased odds of ETW than those who did not. The model did not show any issues of multicollinearity or influential values.

**Table 3:** Multivariable logistic regression analysis of factors associated with ETW

Risk indicators	OR	95% CI	*p*-value
Age			
18–34 years	-	-	
35–54 years	1.242	1.021–1.512	0.030*
≥ 55 years	1.011	0.828–1.234	> 0.9
Sex			
female	-	-	
male	1.435	1.234–1.670	< 0.001*
SDI			
≤ 25th	-	-	
26th – 50th	1.141	0.941–1.384	0.2
51st – 75th	1.090	0.905–1.314	0.4
> 75th	1.207	0.932–1.565	0.2
GERD			
No	-	-	
Yes	1.176	0.953–1.450	0.13
Eating disorder			
No	-	-	
Yes	1.901	1.149–3.208	0.014*
Sleep apnea			
No	-	-	
Yes	0.946	0.729–1.226	0.7
Dry mouth			
No	-	-	
Yes	1.106	0.941-1.300	0.2
Parafunctional habits			
No	-	-	
Yes	1.436	1.235–1.670	< 0.001*
Toothbrushing frequency			
at least once daily	-	-	
twice or more daily	1.141	0.983–1.324	0.083
Medication			
No	-	-	
Yes	0.965	0.829–1.124	0.7
Consumption of alcoholic beverages			
No	-	-	
Yes	1.156	0.993–1.345	0.061

- For each variable, the reference level is denoted by the absence of values

* *p* ≤ 0.05 indicates statistical difference

## Discussion

This retrospective study identified a significant association between age, sex, parafunctional habits (clenching, grinding or bruxism), and eating disorders (bulimia and/or anorexia) with the occurrence of ETW in patients from a College of Dentistry in the Midwest U.S. The findings provide exploratory evidence supporting the potential influence of sociodemographic, behavioral, and health-related factors on ETW occurrence in this population.

Compared with the younger age group (18–34 years), adults aged 35–54 years exhibited higher odds of presenting ETW. This is consistent with reports showing that cumulative exposure to dietary and/or intrinsic acids, such as in patients with reflux or vomiting, and parafunctional habits and other mechanical forces, contribute to progressive tooth wear over time [6]. Additionally, aging renders enamel and dentin more susceptible to erosive wear due to changes in the mineral phase of enamel [10] and a reduction in enamel proteins [11], making the dentition more vulnerable to progression of erosive tooth wear [12]. Interestingly, adults aged ≥ 55 years did not demonstrate a higher odds ratio compared to the 18–34 years group. This finding, however, should be interpreted with caution rather than taken as evidence of lower susceptibility in older adults. Survivor bias may partly explain this pattern, as the most severely affected teeth in older adults may have already been extracted and replaced with prosthetic restorations, which are not susceptible to ETW. Restorative masking may further contribute to this phenomenon, as previously eroded surfaces restored with direct or indirect restorations would not be captured as active ETW in the clinical exam. Cohort effects cannot be excluded either, as older adults may have grown up in an era with different dietary patterns and lower acidic beverage consumption compared to younger generations, resulting in a genuinely lower erosive exposure over their lifetime. Additionally, behavioral adaptations in older adults, such as dietary modifications driven by gastrointestinal conditions or dental sensitivity, may have reduced ongoing acid exposure. Taken together, these factors suggest that the absence of increased odds in the ≥ 55 years group likely reflects the inherent limitations of retrospective record-based designs in capturing the full clinical picture of ETW in older populations.

Male patients were more likely to present ETW, which is consistent with findings across other studies [7, 9, 13, 14]. This association may be related to behavioral or biological differences. However, these factors were not directly assessed in the present study. Previous literature suggests that higher intake of acidic beverages [15, 16] and greater mechanical forces may contribute to increased tooth wear in males [17], but these mechanisms remain speculative in this context. Other proposed explanations, including differences in enamel structure [18] or genetic factors [19, 20], also have been reported. Therefore, the observed sex difference should be interpreted as an association rather than an explanatory relationship. While such mechanisms are promising avenues of explanation, it is important to acknowledge that dietary intake was not directly captured in this study, representing a central limitation. Since differences in acidic food and beverage consumption between males and females might partially explain the observed sex disparity in ETW, the absence of validated dietary data precludes definitive conclusions regarding the relative contribution of nutritional factors to this and other associations reported herein. Future studies should integrate validated dietary questionnaires to clarify the role of nutrition in ETW risk among U.S. populations.

In the current study, the indicator “tooth clenching, grinding or bruxism” was associated with ETW, which is consistent with previous reports suggesting a synergistic interaction between chemical erosion and mechanical wear [21, 22]. Chemical degradation may increase the susceptibility of dental tissues to mechanical forces, thereby accelerating tissue loss [22]. However, the link between these self-reported parafunctional activities and clinical outcomes remains uncertain, as the diagnosis of bruxism should rely on objective methods such as polysomnography rather than solely on patients’ self-reported symptoms [23].

The identification of bulimia and anorexia nervosa as significant risk indicators further supports the established link between eating disorders and ETW, largely due to intrinsic acid exposure from recurrent vomiting [24]. Our findings corroborate a recent meta-analysis reporting that 54.4% of individuals with bulimia and 26.7% with anorexia exhibit dental erosion, with self-induced vomiting increasing the risk 16-fold [25]. Although the prevalence of eating disorders was low in the present cohort, the strength of the association underscores their clinical relevance, particularly given the chronic oral health deterioration and the functional, esthetic, and financial burdens that accumulate over time [26]. Despite the well-established relationship between eating disorders and oral diseases, oral health remains under-addressed in routine eating disorder care, highlighting the need for structured interprofessional collaboration between dental and medical providers to improve early detection, patient education, and long-term outcomes [27, 28].

In contrast, no significant associations were observed for gastroesophageal reflux disease (GERD), alcohol consumption, or dry mouth, despite their established relevance [7]. These findings should be interpreted with caution, as they may reflect limitations in exposure ascertainment, including measurement error related to self-reported data. Information bias, encompassing recall bias, misinterpretation of questions, and social desirability bias, is recognized as one of the most common sources of bias affecting the validity of health research [29]. For example, patients may underreport GERD symptoms or fail to recognize their significance, leading to false negatives. Alcohol consumption is particularly susceptible to social desirability bias, whereby participants tend to underreport socially sensitive behaviors. Similarly, mild/moderate dry mouth symptoms may go unrecognized in the absence of a formal clinical diagnosis, as patients often perceive low-grade xerostomia as transient discomfort rather than a pathological condition. Additionally, residual confounding cannot be ruled out, as unmeasured or imprecisely measured factors may have influenced the observed associations. Therefore, these results should be considered inconclusive rather than indicative of no association.

The SDI, a composite index reflecting area-level deprivation through seven demographic and socioeconomic indicators (e.g., poverty, education, single-parent households, overcrowding, unemployment), did not show association with ETW in this study. While this could reflect true lack of effect, it is also possible that the clinic-based population did not capture the full range of socioeconomic variation or that individual-level socioeconomic measures may be more sensitive than area-level indices.

This study benefits from a large sample size and systematic data extraction from electronic dental records, strengthening the robustness of the regression analysis. The setting of the University of Iowa College of Dentistry further supports the representativeness of findings, as the institution serves as a referral center for the Midwest, treating patients from diverse backgrounds across multiple cities and neighboring states, which broadens generalizability beyond a single local population.

Nevertheless, several limitations should be acknowledged. ETW lesions were identified through diagnostic codes and clinical notes rather than a standardized, validated assessment tool. As a retrospective record review conducted across multiple provider levels, including dental students, graduate residents, and faculty, no uniform protocol or calibration procedure was employed, and validated indices such as the Basic Erosive Wear Examination (BEWE) were not used. This introduces the potential for non-differential misclassification, with mild ETW cases likely being underdiagnosed. Such misclassification would be expected to attenuate the observed associations toward the null, suggesting that the true relationships between ETW and the investigated factors may be stronger than those reported. Also, a complete-case analysis was performed, including only participants with fully completed questionnaires (*n* = 3,089). Although the final sample remained large, this approach may have introduced selection bias if the probability of missing data was related to participant characteristics or outcomes. As the missingness mechanism was not formally evaluated, findings should be interpreted with caution regarding their generalizability. Finally, as a retrospective record review, temporal and causal relationships could not be established. Multicenter studies would be needed to confirm these findings on a broader national scale.

## Conclusions

Within the limits of the present study, age (35–54 years), male sex, parafunctional habits (clenching, grinding or bruxism), and eating disorders (bulimia and/or anorexia nervosa) were associated with the occurrence of ETW. Dental professionals should prioritize early identification and counseling for patients at higher risk, particularly midlife adults, men, bruxers, and individuals with eating disorders. Preventive interventions, closer monitoring, and timely management in these groups could help reduce the long-term impact of ETW.

Future research should employ prospective cohort designs with standardized ETW scoring, incorporate validated dietary and lifestyle measures, and evaluate the incidence and determinants of ETW across diverse U.S. populations. Interprofessional collaboration, especially with medical and mental health providers, will be key in addressing systemic conditions such as eating disorders and GERD that contribute to ETW risk.

## Data Availability

The datasets generated and/or analyzed during the current study are available from the corresponding author upon reasonable request due to privacy or ethical restrictions.
